# Supplementary material for: Juvenile cleaner fish can socially learn the consequences of cheating
Source: Nat Commun. 2020 Mar 3;11:1159. doi: 10.1038/s41467-020-14712-3 (PMC7054547; doi:10.1038/s41467-020-14712-3)
Supplement: Supplementary file 1 — Supplementary Information [file 41467_2020_14712_MOESM1_ESM.pdf]

Supplementary Information

Juvenile cleaner fish can socially learn the consequences of cheating

Truskanov et al.

This file includes:

Supplementary Figure 1

Supplementary Table 1

Supplementary Note 1 and Supplementary Figure 2

Supplementary Figure 3

Supplementary Discussion

Supplementary References

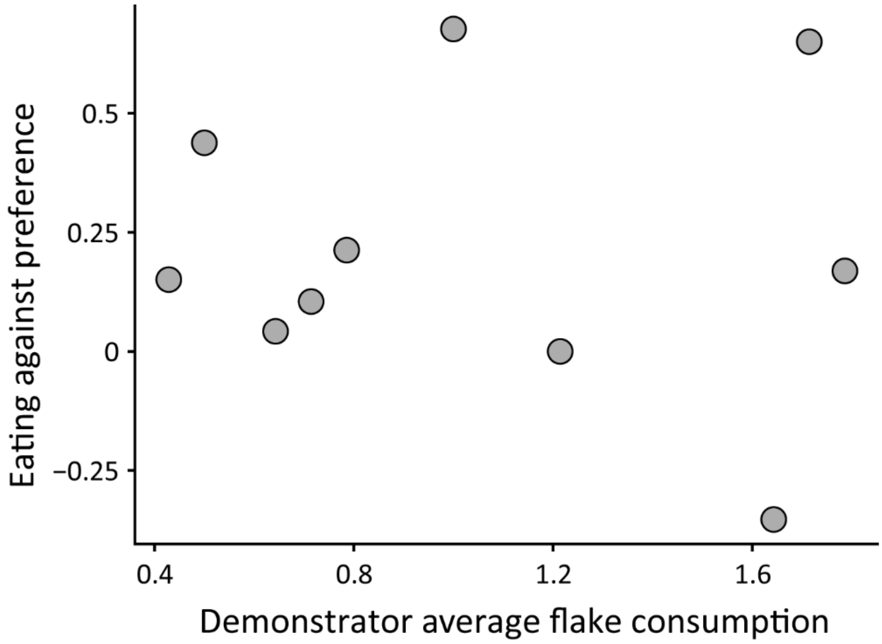

Supplementary Figure 1: Demonstrators' performance does not affect observers' cooperation levels

Adjustment of cooperation levels (extent of eating against preference) by juveniles of the observer treatment in experiment 1, plotted against the average number of flake items consumed by their respective adult demonstrators. N = 10 observer-demonstrator pairs. Source data are provided as a Source Data file.

Supplementary Table 1 – Statistical models testing predictors affecting juvenile cleaners' performance in the 3 social learning experiments.

Models A-B: Linear models analyzing the effects of experimental cohort, treatment group (model A) and demonstrator average flake consumption (model B, relating to cleaners of the observer group only) on juveniles' cooperative feeding choices in the tests of experiment 1. **Model C:** A Generalized linear mixed model (GLMM) with binomial distribution, analyzing the effect of social observation and response type on cleaners' choice of tolerant plates, in the tests of the social learning phase of experiment 2. Fish identity was included as a random intercept. **Model E:** Generalized linear model (GLM) with a quasibinomial distribution, analyzing the effect of treatment group and demonstrators' preference strength on juvenile cleaners' preferences (their choice of demonstrators' preferred vs. un-preferred plates) in the tests of experiment 3. All models included intercept (not shown).

Experiment	Model	N	Dependent variable	Fixed effects	df	χ^2	P
1	A	20	Eating against preference score	Treatment	1	8.797	0.009
				Flake palatability	1	1.097	0.309
	B	10	Eating against preference score (observer treatment only)	Demonstrator average flake consumption	1	0.261	0.625
				Flake palatability	1	0.866	0.383
2	C	19	Choice of tolerant vs. responsive model client	Social observation	1	15.665	>0.0001
				Response type	1	0.011	0.915
				Interaction	1	2.789	0.095
3	D	19	Proportion of choice of demonstrator's preferred color	Treatment	1	0.602	0.438
				Demonstrator's preference score	1	1.162	0.281

Supplementary Note 1

Individual learning about partners' responsiveness to cheating

In experiment 2, prior to the beginning of the social learning experiment, we verified that the juvenile cleaners can use individual learning (IL) to prioritize model clients that are tolerant to cheating over plates that either flee or punish and chase the cleaner for 3s. In each treatment (fleeing/punishing, order counterbalanced), the cleaners were exposed to separate presentations of two model client types differing in colour patterns and responsiveness to cheating. They were then given simultaneous choice tests in which they significantly preferred the tolerant clients (GLHT comparing the juveniles' preferences to a no preference null hypothesis: *IL fleeing*: Z=3.098, P=0.002; *IL punishing*: Z=7.403, P<0.0001. Supplementary Figure 2). This preference was much stronger in the punishing treatment in which the response of the model client was more severe (GLMM: *plate response type:* estimate ± SE=1.216±0.267, N=20, χ^2=20.795, P<0.0001, R^2=0.15. Supplementary Figure 2). Interestingly, this same variable did not seem to affect cleaners' preferences in the social learning phase of our experiment. This result may indicate that when punishment is observed rather than being experienced first-hand during training, its retaliatory effect is reduced. However, as the order of the experiments was fixed, we cannot rule out the possibility that this reduced effect is also due to a gradual habituation to the experimental setup.

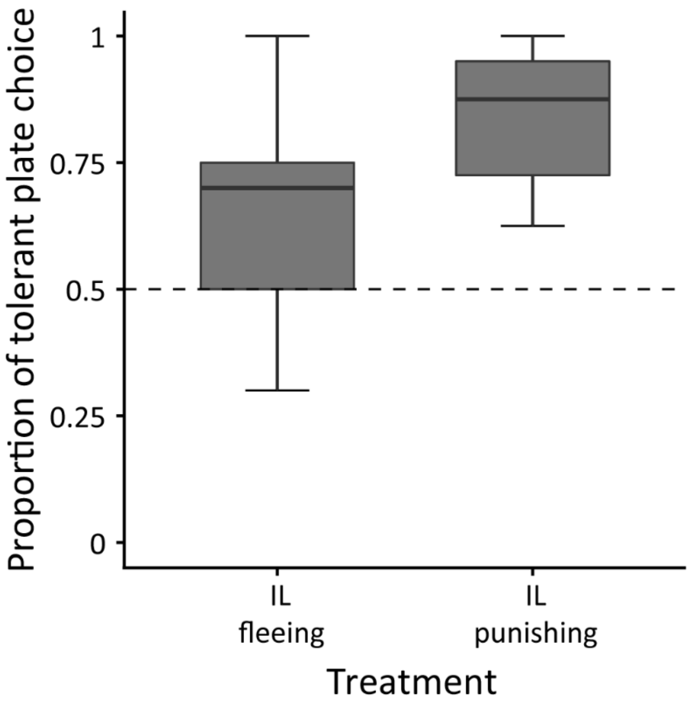

Supplementary Figure 2: Individually learned preference for tolerant clients

Juvenile cleaners' preference for the tolerant (non-responsive) plates in the individual learning phase of experiment 2. Dashed lines mark the preference score expected at random (0.5). N=19 individuals that participated in both treatments (order counterbalanced). Boxplots show the median and interquartile range and whiskers denote 1.5×interquartile range. Source data are provided as a Source Data file.

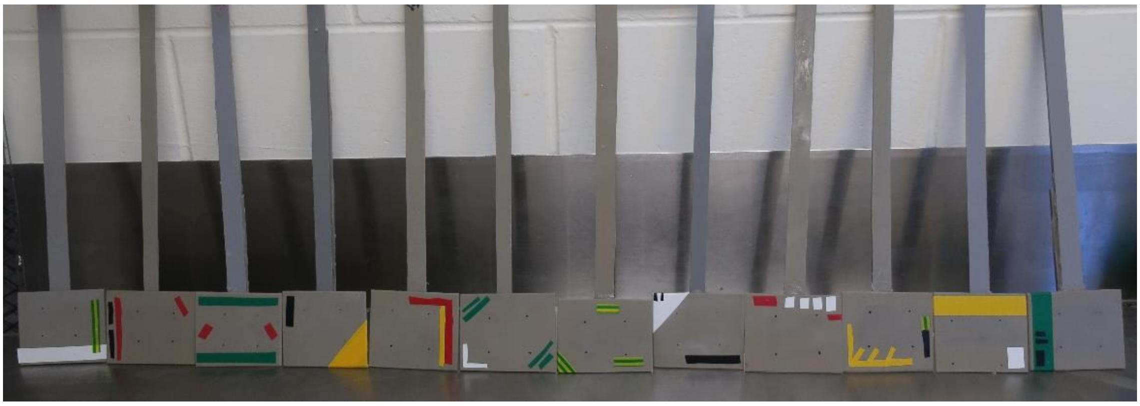

Supplementary Figure 3. Patterns and colors of the client models used in experiment 2

In experiment 2, the plates used in the different treatments were similar in shape and size (7 x 9 cm), but visually distinct, containing unique combinations of different patterns and colors (white, yellow, black, red and green). In each of the days of the experiment, we used a novel pair of plexiglass plate markings, thus comprising the 12 distinct plates shown in the image. Plate pairs are ordered from left to right, according to the experimental day in which they were used. In each day, plate roles were counterbalanced between individuals.

Supplementary Discussion

Social learning about negative consequences and inhibition of prepotent responses

While current knowledge of the use of social learning in cooperative contexts is limited, animals can certainly learn socially about negative consequences in various other contexts. Eavesdropping on the outcomes of conspecifics' mating or competitive interactions is common[1-3], and can help individuals avoid interactions that are likely to yield negative outcomes. For instance, female Japanese quails that observe male-male competition involving high levels of aggression, learn to choose "loser" males, who are less likely to injure them during courtship[4, 5]. Social learning about the risk of predation or its aftermath is also common and can facilitate predator recognition and lead to both avoidance and mobbing behaviours (reviewed by [6, 7]). In addition, social learning about the unpalatability or aversiveness of food, can cause shifts in dietary preferences (e.g.[8-11]). Thus, from this perspective, it is perhaps not surprising that juvenile cleaners could extract information about the negative consequences associated with conspecifics' non-cooperative feeding choices and adjust their behaviour accordingly. Nonetheless, from a mechanistic point of view, the juveniles' social learning was also to some extent complex. Contrary to the latter examples, in experiment 1, the fish did not learn socially to express an innate avoidance response or to divert their dietary preferences towards more palatable options, but rather to inhibit their prepotent responses by targeting less preferred food items, a task that is seemingly more challenging. Cleaners might be particularly successful in such tasks due to their unique ecology, in which selectively feeding against preference is an inherent part of interactions with clients (cleaners also outperform other fish species in individual learning tasks inspired by this ecologically relevant challenge[12]).

Why didn't juveniles copy demonstrators' choices?

Copying of observed behaviours and/or preferences is a widespread phenomenon, occurring in a range of species and contexts[13, 14]. However, in both of the experiments in which this was possible, the juveniles refrained from copying adults. In experiment 1, juveniles foraging behavior was not affected by the adults' demonstrated flake consumption. In experiment 3, juveniles did not copy adults' plate preferences.

Regarding the first result, we note that ectoparasites are cryptic, making it unlikely that juveniles can track directly the precise food being consumed (clients' jolts in response to cleaners' biting of the mucus, can instead serve as proxies to cleaners' feeding choices). Thus, client responses may be a more salient feature of such interactions. Regarding the third experiment, we propose several, not mutually exclusive, explanations for this finding. First, juveniles may use social information selectively[15, 16], only relying on it to guide their decisions when doing so has tangible outcomes in terms of the payoffs obtained. Second, it is possible that the information provided in the first two experiments, i.e. about negative responses of model clients, is more salient than information about conspecifics' choices, and thus more likely to elicit a learned response. Finally, the explanation may be rooted in our experimental design, whereby not seeing the food itself may have caused social leaning to be hindered (see Fig. 4a. Adults' performance during demonstrator training indicates that individual learning is clearly possible in this case, also see[17]). Note, however, that experiments on other species show that the effect of food visibility on social learning doesn't necessarily have to be positive[18, 19], and that the juveniles also didn't seem to be affected by adults' preferences in experiment 1, in which the food was visible.

Supplementary References

1. Danchin E, Giraldeau LA, Valone TJ, Wagner RH. Public information: From nosy neighbors to cultural evolution. *Science* **305**, 487-491 (2004).

2. McGregor PK. *Animal communication networks*. Cambridge University Press (2005).

3. Valone TJ. From eavesdropping on performance to copying the behavior of others: a review of public information use. *Behav Ecol Sociobiol* **62**, 1-14 (2007).

4. Ophir AG, Galef Jr BG. Female Japanese quail that 'eavesdrop'on fighting males prefer losers to winners. *Anim Behav* **66**, 399-407 (2003).

5. Ophir AG, Galef Jr BG. Sexual experience can affect use of public information in mate choice. *Anim Behav* **68**, 1221-1227 (2004).

6. Griffin A. Social learning about predators: a review and prospectus. *Anim Learn Behav* **32**, 131-140 (2004).

7. Crane AL, Ferrari MC. Social learning of predation risk: a review and prospectus. In: *Social learning theory: phylogenetic considerations across animal, plant, and microbial taxa*,(Editor). Nova Science Publisher (2013).

8. Fryday SL, Greigsmith PW. The effects of social-learning on the food choice of the house sparrow (*Passer-domesticus*). *Behaviour* **128**, 281-300 (1994).

9. Van de Waal E, Borgeaud C, Whiten A. Potent social learning and conformity shape a wild primate's foraging decisions. *Science* **340**, 483-485 (2013).

10. Landová E, Hotová Svádová K, Fuchs R, Štys P, Exnerová A. The effect of social learning on avoidance of aposematic prey in juvenile great tits (Parus major). *Anim Cogn* **20**, 855-866 (2017).

11. Thorogood R, Kokko H, Mappes J. Social transmission of avoidance among predators facilitates the spread of novel prey. *Nature ecology & evolution* **2**, 254 (2018).

12. Gingins S, Bshary R. The cleaner wrasse outperforms other labrids in ecologically relevant contexts, but not in spatial discrimination. *Anim Behav* **115**, 145-155 (2016).

13. Galef BG, Giraldeau LA. Social influences on foraging in vertebrates: causal mechanisms and adaptive functions. *Anim Behav* **61**, 3-15 (2001).

14. Hoppitt W, Laland KN. Social processes influencing learning in animals: A review of the evidence. *Adv Stud Behav* **38**, 105-165 (2008).

15. Laland KN. Social learning strategies. *Learn Behav* **32**, 4-14 (2004).

16. Kendal RL, Boogert NJ, Rendell L, Laland KN, Webster M, Jones PL. Social learning strategies: Bridge-building between fields. *Trends Cogn Sci* **22**, 651-665 (2018).

17. Pretot L, Bshary R, Brosnan SF. Factors influencing the different performance of fish and primates on a dichotomous choice task. *Anim Behav* **119**, 189-199 (2016).

18. Truskanov N, Lotem A. The importance of active search for effective social learning: an experimental test in young passerines. *Anim Behav* **108**, 165-173 (2015).

19. Truskanov N, Shy R, Lotem A. Context-specific learning and its implications for social learning. *Behav Ecol* **29**, 1046-1055 (2018).
