# Supplementary material for: Juvenile cleaner fish can socially learn the consequences of cheating
Source: Nat Commun. 2020 Mar 3;11:1159. doi: 10.1038/s41467-020-14712-3 (PMC7054547; doi:10.1038/s41467-020-14712-3)
Supplement: Supplementary file 3 — Reporting Summary [file 41467_2020_14712_MOESM3_ESM.pdf]

nature research

Corresponding author(s): Noa Truskanov

Last updated by author(s): Jan 11, 2020

Reporting Summary

Nature Research wishes to improve the reproducibility of the work that we publish. This form provides structure for consistency and transparency in reporting. For further information on Nature Research policies, see Authors & Referees and the Editorial Policy Checklist .

Statistics

For all statistical analyses, confirm that the following items are present in the figure legend, table legend, main text, or Methods section.

n/a	Confirmed	
☐	☒	The exact sample size (*n*) for each experimental group/condition, given as a discrete number and unit of measurement
☐	☒	A statement on whether measurements were taken from distinct samples or whether the same sample was measured repeatedly
☐	☒	The statistical test(s) used AND whether they are one- or two-sided<br>*Only common tests should be described solely by name; describe more complex techniques in the Methods section.*
☐	☒	A description of all covariates tested
☐	☒	A description of any assumptions or corrections, such as tests of normality and adjustment for multiple comparisons
☐	☒	A full description of the statistical parameters including central tendency (e.g. means) or other basic estimates (e.g. regression coefficient) AND variation (e.g. standard deviation) or associated estimates of uncertainty (e.g. confidence intervals)
☐	☒	For null hypothesis testing, the test statistic (e.g. *F*, *t*, *r*) with confidence intervals, effect sizes, degrees of freedom and *P* value noted<br>*Give P values as exact values whenever suitable.*
☒	☐	For Bayesian analysis, information on the choice of priors and Markov chain Monte Carlo settings
☒	☐	For hierarchical and complex designs, identification of the appropriate level for tests and full reporting of outcomes
☐	☒	Estimates of effect sizes (e.g. Cohen's *d*, Pearson's *r*), indicating how they were calculated

Our web collection on statistics for biologists contains articles on many of the points above.

Software and code

Policy information about availability of computer code

Data collection	No software was used to collect data
Data analysis	No software was used to collect data

For manuscripts utilizing custom algorithms or software that are central to the research but not yet described in published literature, software must be made available to editors/reviewers. We strongly encourage code deposition in a community repository (e.g. GitHub). See the Nature Research guidelines for submitting code & software for further information.

Data

Policy information about availability of data

All manuscripts must include a data availability statement. This statement should provide the following information, where applicable:

- Accession codes, unique identifiers, or web links for publicly available datasets
- A list of figures that have associated raw data
- A description of any restrictions on data availability

The data that support the findings of this study are deposited in Figshare data repository: DOI 10.6084/m9.figshare.8068280.v1 [https://doi.org/10.6084/m9.figshare.8068280.v1]67. Source data for Figures 2-4 and Supplementary Figures 1-2 are provided in a Source Data file.

Field-specific reporting

Please select the one below that is the best fit for your research. If you are not sure, read the appropriate sections before making your selection.

☒ Life sciences ☐ Behavioural & social sciences ☐ Ecological, evolutionary & environmental sciences

Life sciences study design

All studies must disclose on these points even when the disclosure is negative.

Sample size	Sample size was determined based on previous experience with the study system (and constraints of the experimental setup in the field station).
Data exclusions	No data were excluded
Replication	All methods are described in detail to facilitate replication.
Randomization	In experiment 1, individual allocation into treatments was done in a semi-randomized, counterbalanced manner (by rmeasuring individuals' initial food preferences, ranking them based on these preferences, and randomly splitting individuals with similar ranks into the two treaments). In all other experiments, allocation was completely random.
Blinding	Blinding was not possible, since in order to run the experiments, the experimenter had to know to which treatment each individual belonged.

Reporting for specific materials, systems and methods

We require information from authors about some types of materials, experimental systems and methods used in many studies. Here, indicate whether each material, system or method listed is relevant to your study. If you are not sure if a list item applies to your research, read the appropriate section before selecting a response.

Materials & experimental systems

n/a	Involved in the study
☒	☐ Antibodies
☒	☐ Eukaryotic cell lines
☒	☐ Palaeontology
☐	☒ Animals and other organisms
☒	☐ Human research participants
☒	☐ Clinical data

Methods

n/a	Involved in the study
☒	☐ ChIP-seq
☒	☐ Flow cytometry
☒	☐ MRI-based neuroimaging

Animals and other organisms

Policy information about studies involving animals; ARRIVE guidelines recommended for reporting animal research

Laboratory animals	The study did not involve laboratory animals.
Wild animals	40 bluestreak cleaner wrasse participated in our experiments: 20 adults and 20 juveniles (sample size was reduced to 19 in some of the experiments, due to the death of one of the subjects). All fish were caught in the reefs surrounding Lizard island research station using hand nets, and transported directly to the lab. At the end of the study, the fish were released back into their original habitats.
Field-collected samples	The study did not involve collection of samples.
Ethics oversight	Ethics approval was provided by DAFF Animal Ethics Committee (AEC), Queensland Australia.

Note that full information on the approval of the study protocol must also be provided in the manuscript.
